# Effects of diet hulless barley and beta-glucanase levels on ileal digesta soluble beta-glucan molecular weight and carbohydrate fermentation in laying hens

**DOI:** 10.1016/j.psj.2022.101735

**Published:** 2022-01-18

**Authors:** Namalika D. Karunaratne, Henry L. Classen, Nancy P. Ames, Michael R. Bedford, Rex W. Newkirk

**Affiliations:** ⁎ Department of Animal and Poultry Science, University of Saskatchewan, Saskatoon, S7N 5A8, Saskatchewan, Canada; † Agriculture and Agri-Food Canada, Winnipeg, R3T 2E1, Manitoba, Canada; ‡ AB Vista, Marlborough, Wiltshire, SN8 4AN, United Kingdom

**Keywords:** fermentation, prebiotic, pH, feed enzyme, β-glucan

## Abstract

Exogenous β-glucanase (**BGase**) improves nutrient digestibility and production performance in laying hens fed barley-based diets, but the effect of enzyme and the dosage on β-glucan depolymerization and fermentation in the gastrointestinal tract is poorly understood. The objectives of the study were to determine the effects of hulless barley (**HB**) and BGase levels on digestive tract β-glucan depolymerization and fermentation in laying hens. A total of 108 Lohman-LSL Lite hens were housed in cages and fed 2 levels of HB (CDC Fibar; 0 and 73%) by substituting wheat in the diet and graded levels of BGase (Econase GT 200 P from ABVista; 0, 0.01 and 0.1% – 0, 20,000, and 200,000 BU/kg) in a 2 × 3 factorial arrangement. Birds were fed experimental diets for 8 weeks, starting at 35 wk of age. Digestive tract samples were collected at the end of the experiment. Statistical significance was set at *P* ≤ 0.05. Beta-glucan peak molecular weight was lower with the 0.1 compared to both 0 and 0.01% BGase levels, whereas weight average molecular weight was lower with the 0.1 compared to 0% BGase for 73% HB. The maximum molecular weight for the smallest 10% β-glucan molecules decreased with the increasing BGase. Overall, β-glucan molecular weight in the ileum was higher when the birds were given 73 in comparison to 0% HB diets. Total and major short chain fatty acids (**SCFA**) in the ileum were lower with 0.1 and 0.01 (except propionic acid) compared to 0% BGase in the birds fed 73% HB, but not 0% HB. Interactions between the main effects were found for the cecal acetic and isobutyric acids. In conclusion, exogenous BGase depolymerized high molecular weight β-glucan in HB and wheat. The effects of HB and BGase on carbohydrate fermentation were not apparent, although it appears ileal SCFA concentrations were lower with increasing levels of BGase.

## INTRODUCTION

Soluble β-glucan in barley is concentrated in the endosperm cell walls of the kernel (McNab and Smithard, 1992; Izydorczyk and Dexter, 2008) and increases digesta viscosity in the small intestine of chickens (Classen et al., 1985; Salih et al., 1991). However, adding exogenous β-glucanase (**BGase**) to barley-based diets can reduce the digesta viscosity and increase nutrient digestibility, egg production, and feed efficiency in laying hens (Jeroch, 1991; Brenes et al., 1993; Lázaro et al., 2003a).

The ceca is the main site of fermentation in the digestive tract of chickens due to a more diverse bacterial population (Salanitro et al., 1974). The access to the ceca is restricted to very fine particulate and less viscous soluble molecules (Choct et al., 1996; Svihus et al., 2013). Barley β-glucan has a high molecular weight (Cui et al., 2000; Storsley et al., 2003; Biliaderis and Izydorczyk, 2007), and this might affect the ability of β-glucan to enter the ceca. Therefore, it is crucial to study the effect of exogenous BGase on depolymerizing barley β-glucan into low molecular weight carbohydrates. Diet supplementation of non-starch polysaccharidases increases cecal fermentation while reducing the ileal fermentation of carbohydrates, which supports the increased ability of fiber to enter the ceca with the use of enzymes (Choct et al., 1996, 1999). Further, low molecular weight arabinoxylan in wheat increased the beneficial microbial population in chicken ceca (Courtin et al., 2008), indicating the alteration of carbohydrate fermentation in chickens. However, modification of the microbiota and carbohydrate fermentation in the ceca might be related to the increased entry of low molecular weight carbohydrates into the site of fermentation, or the preference of microbiota for the low versus the high molecular weight carbohydrates (Lei et al., 2012). Therefore, it is logical to investigate whether the effects noted with arabinoxylan and arabinoxylo-oligosaccharides can be replicated with β-glucan by feeding a high β-glucan barley with increasing levels of BGase to determine if β-glucan content or its molecular weight within the ceca can influence fermentation.

The effect of dietary enzyme use in barley-based diets on carbohydrate fermentation has been studied in broiler chickens (Józefiak et al., 2005, 2006). Enzyme either increased or had no effect on short chain fatty acids (**SCFA**) levels in the crop and caeca and did not affect ileal levels. Similar studies have not been performed in laying hens. The degree and the exact location of carbohydrate fermentation in the gastrointestinal tract of laying hens might differ from broiler chickens since laying hens are older and have a more mature digestive tract and complex gastrointestinal microbial population (Videnska et al., 2014). Moreover, the high calcium content in laying hen diets compared to broiler diets may influence the pH of the digesta content due to its high buffering capacity. Interpretation of the effects of enzyme use in barley diets is confounded by the use of both β-glucanase and xylanase in previous research (Brenes et al., 1993; Mathlouthi et al., 2003; Józefiak et al., 2005, 2006). Consequently, substrates for fermentation would include low molecular weight carbohydrates derived from arabinoxylan and β-glucan (Bach Knudsen, 2014). The current study is distinct from the previous research due to the use of a purified form of BGase, which permits evaluation of the single effect of BGase and hulless barley **(HB)** β-glucan on fermentation in chickens. The impact of digestive tract fermentation has practical importance in laying hen flocks as it may influence the digestive tract microbiota and, consequently, bird health and food safety (Ding et al., 2018).

The objectives of the study were to examine the effects of HB and exogenous BGase levels on ileal soluble β-glucan depolymerization and carbohydrate fermentation in laying hens. It was hypothesized that BGase would depolymerize high molecular weight β-glucan in HB, reduce carbohydrate fermentation in the ileum, and increase the cecal fermentation of laying hens.

## MATERIALS AND METHODS

The experimental procedure was approved by the Animal Research Ethics Board of the University of Saskatchewan and adhered to the Canadian Council on Animal Care guidelines for humane animal use (Canadian Council on Animal Care, 1993, 2009).

### Birds and Housing

A total of 108 Lohman-LSL Lite hens were used for the experiment. Day-old Lohman-LSL Lite chicks were obtained from a commercial hatchery and raised on litter floor pens at the Poultry Centre of the University of Saskatchewan, SK, Canada. Pullet rearing, feeding, lighting, and other environmental conditions approximated Lohman recommendations (Lohman-LSL Lite Management Guide, 2013). Pullets were transferred to Layer Specht conventional cages (60.96 cm length, 39.37 cm width, and 40 cm height: 502.75 cm^2^/bird) in an environmentally controlled barn at 18 wk of age. Each cage was equipped with a lubing nipple drinker and a feed trough along the length of the cage. Barn temperature was maintained at approximately 21°C, and hens were provided with 16 h of light per day at a light intensity of 10 lux at the feeder level. Hens were fed a commercial laying hen ration that met Lohman recommendations until the start of the experiment at 35 wk of age, after which experimental diets were fed for 8 wk. Dietary treatments were randomly assigned to individual cages (6 cages per treatment), housing 3 birds per cage.

### Experimental Diets

The experiment was designed as a 2 × 3 factorial arrangement with 2 levels of HB (CDC Fibar; β-glucan – 8.0%; 0 and 73%) by substituting wheat in the diet and 3 levels of BGase (Econase GT 200 P from ABVista, Wiltshire, UK; 0, 0.01 and 0.1%) calculated to provide 0, 20,000, and 200,000 BU/kg, respectively, of enzyme activity. The industry standard for the use of this BGase in poultry diets is 0.01%. The enzyme was originated from a genetically modified strain of *Trichoderma reesei* and it contains a declared minimum BGase activity of 200,000 BU (β-glucanase units)/g. Diets were formulated to meet Lohman-LSL Lite specifications (Lohman-LSL Lite Management Guide, 2013), and were balanced for AME and digestible amino acids. Amino acid content was determined according to AMINODat 5.0 nutritional database of feed ingredients. Hulless barley and wheat were assumed to have approximately the same nutrient composition during feed formulation. The diets were fed in pellet form and the pelleting temperature was 68 to 70°C to minimize high temperature induced BGase inactivation. Feed and water were given ad-libitum before the trial start and throughout the study period. Dietary composition and calculated nutrient levels are shown in Table 1. The average BGase activity for 0% HB- and 73% HB-based diets were: 0% BGase, 8,938 U/kg; 0.01% BGase, 17,428 U/kg; and 0.1% BGase, 93,092 U/kg. The analyzed enzyme activity mirrored the addition of enzymes to the diet but was not the same. The level of enzyme activity in the 0% BGase diets likely represents endogenous enzyme activity, while reduced values at 0.01 and 0.1% may indicate enzyme loss during feed processing. The enzyme levels mirrored the planned values and are assumed to meet the objectives of the current research. Further, xylanase activity was nondetectable in all the diets (<2,000 U/kg).

**Table 1:** Ingredients and calculated nutrient levels of experimental diets.

Ingredient	Quantity (%, as fed basis)
Cereal grain (wheat or hulless barley)	72.56
Soybean meal	14.13
Canola oil	1.79
Mono-dicalcium phosphate	1.09
Limestone	9.38
Sodium chloride	0.31
Vitamin-mineral premix^1^	0.50
Choline chloride	0.10
Rovimix HYD 62.5	0.04
DL-Methionine	0.09
L-Lysine HCl	0.01
Nutrient, calculated	
AME (kcal/kg)	2,800
Dry matter	93.45
Crude protein	16.04
Crude fat	3.34
Calcium	3.73
Chloride	0.28
Non-phytate phosphorous	0.40
Potassium	0.61
Sodium	0.16
Linoleic acid	1.18
Digestible arginine	0.82
Digestible isoleucine	0.51
Digestible leucine	1.00
Digestible lysine	0.61
Digestible methionine	0.30
Digestible methionine and cysteine	0.56
Digestible threonine	0.46
Digestible tryptophan	0.18
Digestible valine	0.62

1 Vitamin-mineral premix provided the following per kilogram of complete diet: vitamin A (retinyl acetate + retinyl palmitate), 8,000 IU; vitamin D_3_, 3,000 IU; vitamin E (dl-α-tocopheryl acetate), 25 IU; menadione, 1.5 mg; thiamine, 1.5 mg; riboflavin, 5.0 mg; niacin, 30 mg; pyridoxine, 1.5 mg; vitamin B_12_, 0.012 mg; pantothenic acid, 8.0 mg; folic acid, 0.5 mg; biotin, 0.06 mg; copper, 5 mg; iron, 80 mg; manganese, 80 mg; iodine, 0.8 mg; zinc, 80 mg; selenium, 0.3 mg; calcium carbonate, 500 mg; ethoxyquin, 0.625 mg; wheat middlings, 3,822.79 mg.

### Sample Collection

At the end of the experiment (43 wk of age), all the hens were euthanized by intravenous administration of T61 containing Embutramide, Mebezonium iodide, and Tetracaine hydrochloride (Merck Animal Health, Kirkland, Quebec, Canada) into the brachial vein. Individual bird weights were recorded. Two birds per cage were used to measure in situ pH values in mid ileal and cecal contents using a Beckman Coulter 34 pH meter (Model PHI 34, Beckman Instruments, Fullerton, CA). Portions of the ileal and cecal contents were collected into plastic centrifuge tubes separately from 2 birds and stored at −20°C until the analysis of SCFA. The rest of the ileal content was pooled and collected into plastic snap-cap vials to analyze β-glucan content. A portion of the pooled ileal content was centrifuged for 5 min at 17,013 × *g* at 40°C using a Beckman microfuge (Model E348720, Beckmann Instruments, INC, Palo Alto, CA). The viscosity of the pooled ileal supernatant was measured using a Brookfield cone-plate digital viscometer (Model LVDV-Ⅲ, Brookfield Engineering Labs, INC, Stoughton, MA), which was maintained at 40°C (40 rpm; shear rate 300 s^−1^). The remainder of the ileal supernatant was stored in plastic micro-centrifuge tubes at −80°C for β-glucan molecular weight distribution analysis. The viscosity of wheat and hulless barley was analyzed according to Scoles et al. (1993) using sodium acetate buffer as the extraction media and a Brookfield cone-plate digital viscometer (Model LVDV-Ⅲ, Brookfield Engineering Labs).

### Nutritional Analysis

Experimental diets and grains (HB and wheat) were ground through 1 mm (CP, fat, ash, and dietary fiber analyses) and 0.5 mm (total starch and β-glucan analyses) screen-hole sizes using a Retsch laboratory mill (Retsch ZM 200, Germany) before chemical analysis. Beta-glucan was analyzed in ingredients, diets, and ileal digesta according to the AOAC Method 995.16, AACC Method 32-23 and ICC Standard Method No. 168 (AOAC, 2006; AACC, 2010; ICC, 2011) using a Megazyme analysis kit (Mixed-linkage beta-glucan assay procedure/McCleary method, Megazyme International Ireland Ltd., Bray Business Park, Bray, Co. Wicklow, Ireland). Moisture was analyzed using method 930.15 of AOAC (2006). The total starch analysis was completed based on the AOAC method 996.11 and the AACC method 76-13.01 (AOAC, 2006; AACC, 2010) using a Megazyme kit (Total starch assay procedure, Amyloglucosidase/α-amylase method, Megazyme International Ireland Ltd., Bray Business Park, Bray, Co.). Nitrogen was analyzed using a Leco protein analyzer (Model Leco-FP-528L, Leco Corporation, St. Joseph, MA), and 6.25 was used as the N to CP conversion factor. Fat content was analyzed by ethyl ether extraction using Goldfish Extraction Apparatus (Labconco model 35001; Labconco, Kansas, MO) following the AOAC method 920.39 (AOAC, 2006). Ash content was determined according to the AOAC method 942.05 (AOAC, 2006) using a muffle oven (Model Lindberg/Blue BF51842C, Asheville, NC). Insoluble dietary fiber (**IDF**) and soluble dietary fiber (**SDF**) were analyzed using a Megazyme kit (Total dietary fiber assay procedure, Megazyme International Ireland Ltd., Bray Business Park, Bray, Co.) according to the AOAC method 991.43 and the AACC method 32-07.01 (AOAC, 2006; AACC, 2010), and total dietary fiber (**TDF**) was calculated by adding the values for SDF and IDF. Experimental diets were analyzed for enzyme activity (BGase and xylanase) using AB Vista methods of ESC Standard Analytical Method SAM042-01 and SAM038, respectively (AB Vista). The BGase and xylanase activity analyses was based on the end-point determination of reducing sugars using a DNS-based colorimetric system using barley β-glucan and birch xylan as substrates, respectively, for BGase and xylanase activity.

### Beta-Glucan Molecular Weight Distribution

Ileal supernatant was analyzed for β-glucan molecular weight distribution using size exclusion chromatography followed by calcofluor post-column detection for fluorescent recognition (Boyd et al., 2017). Ileal supernatant was boiled for 15 min and centrifuged for 10 min at 9,000 × *g* using a Beckman microfuge (Model E348720, Beckmann instruments) before analysis of the samples using HPLC. The 2 columns used for HPLC were a Shodex OHpak SB-806M column with OHpak SB-G guard column and a Waters Ultrahydrogel linear column. Tris buffer (0.1M; pH = 8) was used as the mobile phase. Beta-glucan peak molecular weight (**Mp**) and weight average molecular weight (**Mw**) of each sample were determined using the molar mass distribution curve of the HPLC software. Peak molecular weight is defined as the molecular weight of the highest fraction of β-glucan molecules. The definition of Mw is the average (considering the weight fraction of each molecule type) of the molecular weight of all β-glucan molecules. The maximum molecular weight for the smallest 10% β-glucan molecules (**MW-10%**) was also analyzed based on the molar mass distribution curve.

### Short Chain Fatty Acids Analysis

Short chain fatty acids were analyzed in triplicate according to the procedure described by Zhao et al. (2006) with minor changes. The internal standard for the analysis was made up of 20 mL of 25% phosphoric acid, 300 µL of isocaproic acid and deionized water. Three hundred microliters of acetic acid, 200 µL of propionic acid, 100 µL of butyric acid and 50 µL of isobutyric, isovaleric, valeric, caproic, and lactic acids were used to make the standard solution. Digesta samples were thawed and mixed with 25% phosphoric acid at a 1:1 ratio and kept at room temperature for 10 min with occasional shaking. The mixture was centrifuged at 12,500 × *g* for 10 min. The supernatant (1 mL) was mixed with the internal standard at a 1:1 ratio and centrifuged at 12,500 × *g* for 10 min. The sample was filtered using a 0.45-micron nylon filter, and the filtrate was filled into a gas chromatography autosampler vial and injected into the Zebron Capillary Gas chromatography column (length: 30 m, internal diameter: 0.25 mm, film thickness: 0.25 µm; Zebron ZB-FFAP, Phenomenex, Torrance, CA). The SCFA analysis was completed using a Thermo Scientific Gas chromatography system (Model Trace 1310, Milan, Italy).

### Statistical Analysis

The experiment was a complete randomized design. The cage was considered the experimental unit, and there were 6 replications per treatment. Data were analyzed using SAS 9.4 Proc mixed model (SAS, 2008). The level of significance was set at *P* ≤ 0.05. Mean separation was completed using the Tukey-Kramer test.

## RESULTS

### Ingredient and Experiment Diet Nutrient Composition (on DM Basis)

Hulless barley contained 26.3% TDF, 17.4% IDF, 8.8% SDF, and 7.2% total β-glucan. The values for TDF, IDF, SDF, and total β-glucan in wheat were 14.1, 11.9, 2.2, and 0.6%, respectively. In HB, total starch, CP, fat, and ash were determined as 47.2, 15.0, 2.1, and 1.6%, respectively, whereas the same parameters were 57.7, 13.5, 1.2, and 1.5%, respectively, in wheat (Table 2). The analyzed chemical compositions of the treatment diets are shown in Table 3.

**Table 2:** Analyzed nutrient composition of dietary wheat and hulless barley.

Parameter	Quantity (%, as fed basis)	
	Wheat	Hulless barley
Dry matter	87.9	90.6
Total starch	57.7	47.2
Crude protein	13.5	15.0
Ether extract	1.2	2.1
Ash	1.5	1.6
Total dietary fiber	14.1	26.3
Insoluble dietary fiber	11.9	17.4
Soluble dietary fiber	2.2	8.8
Total β-glucan	0.6	7.2

**Table 3:** Analyzed chemical composition of the experiment diets (%, as fed basis).

Parameter	0% Hulless barley			73% Hulless barley		
	0% BGase	0.01% BGase	0.1% BGase	0% BGase	0.01% BGase	0.1% BGase
Dry matter	93.4	93.3	93.5	93.8	94.5	94.2
Total starch	41.0	39.6	39.0	35.4	34.4	35.1
Crude protein	16.5	16.8	17.1	16.9	17.9	18.3
Ether extract	1.6	2.3	2.2	2.4	3.1	3.2
Ash	7.9	9.4	9.0	7.6	7.8	9.3
Total dietary fiber	25.1	26.4	26.2	27.6	28.4	29.4
Insoluble dietary fiber	21.3	22.8	23.3	20.4	19.7	22.3
Soluble dietary fiber	3.8	3.5	2.9	7.1	8.6	7.0
Total β-glucan	0.6	0.5	0.6	5.3	5.8	6.0

### Beta-Glucan Content and β-Glucan Molecular Weight Distribution

An interaction between HB and BGase was not found for ileal β-glucan content in laying hens (Table 4). However, β-glucan content in ileal digesta was higher in the birds fed 73 compared to 0% HB-based diets (14.2 vs. 2.0%). Beta-glucanase did not affect the ileal β-glucan content.

**Table 4:** Effects of hulless barley and β-glucanase on soluble β-glucan molecular weight, β-glucan content, ileal viscosity, and digestive tract pH of laying hens at 43 wk of age.

Parameter	0% HB^1^			73% HB			SEM	Probability		
	0% BGase	0.01% BGase	0.1% BGase	0% BGase	0.01% BGase	0.1% BGase		HB	BGase	HB × BGase
Molecular weight (g/mol)										
Mp^2^	14,210^ab^	12,521^ab^	11,019^b^	19,393^a^	19,864^a^	8,184^b^	1,017.2	0.046	0.001	0.030
Mw	18,174^b^	16,543^b^	19,079^ab^	27,431^a^	20,205^ab^	11,509^b^	1,122.8	0.301	0.004	0.001
MW-10%	5,365	4,113	1,288	7,833	4,502	1,759	442.3	0.042	<0.001	0.198
Total β-glucan content (% of DM)	1.96	2.06	2.14	14.48	14.26	13.90	0.327	<0.001	0.820	0.505
Viscosity (cP)	6.90	6.74	5.77	5.23	4.07	3.36	0.268	<0.001	0.002	0.426
pH										
Ileum	7.03	7.03	7.26	7.34	7.19	7.17	0.050	0.236	0.686	0.265
Ceca	6.14	5.99	6.21	6.18	6.55	6.21	0.069	0.152	0.794	0.188

a-b Means within a row not sharing a common superscript are significantly different (*P* ≤ 0.05).

1 Abbreviations: BGase, β-glucanase; HB, hulless barley; SEM, pooled standard error of mean (n = 6 cages per treatment).

2 Mp = peak molecular weight; Mw = weight average molecular weight; MW-10% = The maximum molecular weight for the smallest 10% molecules.

**Figure 1:**
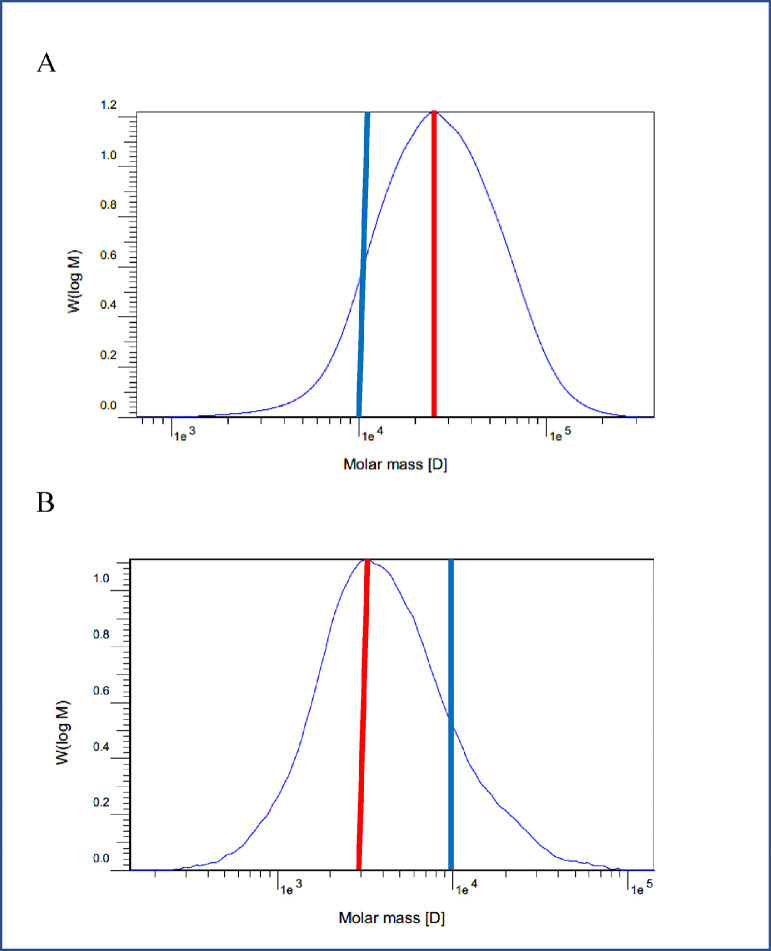
Beta-glucan molecular weight distribution in soluble ileal digesta from 43 wk old laying hens fed 73% hulless barley-based diets. Blue lines denote point 1e^4^ on the x-axis and red lines indicate the Mp of the distribution curve. (A) 0% β-glucanase; (B) 0.1% β-glucanase.

The interactions between the main effects were significant for Mp and Mw of soluble β-glucan in the ileal digesta of laying hens (Table 4). Beta-glucanase did not affect Mp and Mw when the birds were given a 0% HB diet. However, Mp was lower for the 0.1 compared to 0 and 0.01% BGase levels when the hens were fed 73% HB. In addition, Mw was lower for the 0.1 compared to the 0% BGase level in birds fed 73% HB diets; the average Mw for the 0.01% BGase treatment was intermediate. Further, Mw was higher with the 73 than the 0% HB when the birds were not fed BGase. An interaction was not found for the β-glucan MW-10% of ileal digesta. However, it was higher for the 73 (4,698 g/mol) than 0% HB (3,589 g/mol) diets and decreased with increasing BGase (0% BGase, 6,599 g/mol; 0.01% BGase, 4,308 g/mol; 0.1% BGase, 1,524 g/mol). The effect of BGase on molecular weight change in 73% HB diets is shown in Figures 1A and 1B.

### Viscosity

Laying hen ileal soluble digesta viscosity was not affected by the interaction between HB and BGase levels (Table 4). However, the main effects were significant with viscosity levels lower for the 73 (4.22 cP) than the 0% (6.47 cP) HB level and viscosity decreasing with increasing BGase level; viscosity was lower for the 0.1 (4.56 cP) than 0% (6.06 cP) treatment, and the 0.01% level (5.41 cP) was intermediate and not different than the lower or higher levels.

### Intestinal pH and Short Chain Fatty Acids

Ileal and cecal pH values were not affected by HB, BGase, or their interaction (Table 4).

Interactions between HB and BGase were found for the ileal concentrations of total and major SCFA (acetic, propionic, butyric, and lactic acids) in laying hens (Table 5). Beta-glucanase level did not affect concentrations of SCFA when the hens were fed 0% HB. However, SCFA values decreased incrementally with increasing BGase levels when the birds were fed 73% HB-based diets. Statistically, 0.1% BGase levels were either lower than the 0% BGase treatment (propionic and butyric acids), or both 0 and 0.01% BGase levels (total, acetic, and lactic acids). The interaction was also significant for caproic acid concentration, but differences were minor and did not follow obvious trends. Isobutyric, valeric, and isovaleric acid levels affected with BGase levels in the diets. Isobutyric acid decreased with increasing BGase levels (isobutyric – 2.6, 0.9, 0 µmol/g, respectively for 0, 0.01, and 0.1% BGase). Valeric acid concentration was lower with 0.01 and 0.1% BGase than 0% BGase, and isovaleric acid concentration was lower with 0.01% BGase than 0% BGase (valeric – 2.6, 1.7, 1.7 µmol/g; isovaleric – 2.6, 1.6, 2.0 µmol/g, respectively for 0, 0.01, and 0.1% BGase levels).

**Table 5:** Effects of hulless barley and β-glucanase on ileal short chain fatty acids of laying hens at 43 wk of age.

Parameter	0% HB^1^			73% HB			SEM	Probability		
	0% BGase	0.01% BGase	0.1% BGase	0% BGase	0.01% BGase	0.1% BGase		HB	BGase	HB × BGase
SCFA (µmol/g of wet ileal content)										
Total SCFA	121.0^b^	120.9^b^	114.5^bc^	138.7^a^	118.1^b^	101.8^c^	1.89	0.801	<0.001	0.0002
Acetic acid	44.6^b^	45.9^b^	44.3^bc^	54.1^a^	45.9^b^	39.1^c^	0.73	0.188	<0.001	<0.001
Propionic acid	17.0^a^	17.0^a^	16.9^a^	18.1^a^	16.5^ab^	14.8^b^	0.21	0.212	0.001	0.002
Butyric acid	8.2^ab^	8.0abc	7.5abc	8.8^a^	7.1^bc^	6.6^c^	0.15	0.144	0.001	0.050
Isobutyric acid	2.5	0.9	0.0	2.7	0.8	0.0	0.15	0.792	<0.001	0.898
Valeric acid	2.5	2.0	1.6	2.6	1.4	1.8	0.11	0.725	0.002	0.166
Isovaleric acid	2.5	1.9	1.8	2.7	1.4	2.2	0.11	0.958	0.002	0.268
Caproic acid	1.0^a^	0.9^ab^	0.9^ab^	1.1^a^	0.6^b^	0.9^ab^	0.04	0.190	0.003	0.042
Lactic acid	42.3^b^	44.0^ab^	41.2^bc^	48.3^a^	44.0^ab^	36.2^c^	0.68	0.782	<0.001	0.001
Molar percentage of total SCFA										
Acetic acid	36.9^b^	38.0^ab^	38.7^a^	39.0^a^	39.0^a^	38.4^ab^	0.17	0.003	0.217	0.008
Propionic acid	14.0^b^	14.1^b^	14.7^a^	13.1^c^	14.0^b^	14.5^a^	0.07	<0.001	<0.001	0.003
Butyric acid	6.8	6.6	6.6	6.3	6.0	6.5	0.12	0.059	0.558	0.548
Isobutyric acid	2.1	0.6	0.0	1.9	0.6	0.0	0.09	0.707	<0.001	0.899
Valeric acid	2.0	1.7	1.4	1.9	1.1	1.7	0.09	0.542	0.046	0.144
Isovaleric acid	2.0	1.5	1.6	1.9	1.1	2.1	0.09	0.958	0.004	0.080
Caproic acid	0.9^a^	0.8^a^	0.8^a^	0.8^a^	0.5^b^	0.9^a^	0.03	0.131	0.004	0.014
Lactic acid	35.0	36.4	35.9	34.7	37.2	35.5	0.17	0.856	<0.001	0.183

a-c Means within a row not sharing a common superscript are significantly different (*P* ≤ 0.05).

1 Abbreviations: BGase, β-glucanase; HB, hulless barley; EM, pooled standard error of mean (n = 12 birds per treatment).

Main effect interactions were found for the ileal molar percentages of acetic, propionic, and caproic acids (Table 5). Acetic acid percentage was higher with the 0.1% BGase compared to 0% BGase, and propionic acid percentage was higher with the 0.1% BGase compared to 0 and 0.01% BGase at the 0% HB. In addition, the propionic acid percentage increased with the increasing BGase at the 73% HB. The interaction for caproic acid concentration again did not follow meaningful biological trends. Proportional levels of isobutyric, valeric, isovaleric and lactic acids were affected by BGase with a tendency for valeric and isovaleric values to be lowest and lactic acid values to be highest for 0.01% BGase (valeric – 2.0, 1.4, 1.6%; isovaleric – 2.0, 1.3, 1.9%; lactic – 34.9, 36.8, 35.7%, respectively for 0, 0.01 and 0.1% BGase levels). However, the isobutyric acid percentage decreased with the increasing BGase in the diets (0% BGase, 2.0%; 0.01% BGase, 0.6%; 0.1% BGase, 0.0%).

Total cecal SCFA levels were not affected by HB, BGase, or their interaction (Table 6). Interactions between HB and BGase were found for cecal acetic and isobutyric acid concentrations. There was no effect of BGase on these acid concentrations for the 0% HB treatments. However, acetic acid concentration was lower with the 0.1% BGase compared to the 0% BGase in hens fed 73% HB. Further, isobutyric acid concentration was lower with the 0 compared to 0.01 and 0.1% BGase levels when the birds were given 73% HB-based diets. The propionic acid concentration was higher for the 0% BGase (72.8 µmol/g) fed birds compared to those fed 0.01 (67.7 µmol/g) and 0.1% (67.1 µmol/g) BGase levels. Isovaleric and caproic acid concentrations were higher when birds were fed 73% HB than those fed 0% HB. The valeric acid concentration was lower for the 0% BGase (4.3 µmol/g) compared to the diets containing 0.01 (9.9 µmol/g) and 0.1% (7.8 µmol/g) BGase. Furthermore, caproic acid concentration was low with the 0% BGase compared to the 0.01% BGase (2.7 vs. 4.2 µmol/g).

**Table 6:** Effects of hulless barley and β-glucanase on cecal short chain fatty acids of laying hens at 43 wk.

Parameter	0% HB^1^			73% HB			SEM	Probability		
	0% BGase	0.01% BGase	0.1% BGase	0% BGase	0.01% BGase	0.1% BGase		HB	BGase	HB × BGase
SCFA (µmol/g of wet ileal content)										
Total SCFA	322.3	317.4	323.1	342.9	330.9	304.8	4.25	0.531	0.197	0.138
Acetic acid	194.3^ab^	185.3^ab^	194.9^ab^	209.2^a^	193.7^ab^	178.2^b^	2.59	0.658	0.029	0.024
Propionic acid	71.6	66.5	69.7	74.1	68.9	64.6	0.86	0.953	0.009	0.099
Butyric acid	33.1	31.6	32.4	34.4	33.4	30.2	0.44	0.736	0.072	0.134
Isobutyric acid	9.9^a^	9.9^a^	10.4^a^	5.8^b^	10.2^a^	9.6^a^	0.38	0.027	0.014	0.024
Valeric acid	3.2	9.7	6.9	5.5	10.1	8.7	0.54	0.126	<0.001	0.684
Isovaleric acid	8.0	9.8	5.9	10.1	10.1	9.5	0.41	0.012	0.072	0.234
Caproic acid	1.8	4.2	2.6	3.6	4.3	3.7	0.24	0.031	0.018	0.322
Molar percentage of total SCFA										
Acetic acid	60.3^ab^	58.3^b^	60.3^ab^	61.1^a^	58.4^b^	58.4^b^	0.24	0.409	<0.001	0.022
Propionic acid	22.2	20.9	21.6	21.6	20.8	21.2	0.09	0.031	<0.001	0.540
Butyric acid	10.2	9.9	10.0	10.0	10.0	9.9	0.11	0.253	0.057	0.138
Isobutyric acid	3.0^a^	3.1^a^	3.2^a^	1.5^b^	3.1^a^	3.1^a^	0.03	0.005	0.002	0.001
Valeric acid	0.9	3.0	2.1	1.5	3.0	2.8	0.16	0.114	<0.001	0.480
Isovaleric acid	2.4^ab^	3.1^a^	1.8^b^	2.9^ab^	3.0^a^	3.1^a^	0.12	0.013	0.097	0.050
Caproic acid	0.5	1.3	0.7	1.0	1.3	1.2	0.07	0.033	0.008	0.276

a-b Means within a row not sharing a common superscript are significantly different (*P* ≤ 0.05).

1 Abbreviations: BGase, β-glucanase; HB, hulless barley; SEM, pooled standard error of mean (n = 12 birds per treatment).

The molar percentages of cecal acetic, isobutyric and valeric acid concentrations were affected by the interactions between HB and BGase (Table 6). Acetic acid was higher for 0 compared to 0.01 and 0.1% BGase levels when a 73% HB diet was fed, but the enzyme did not affect proportional values when the birds were fed a 0% HB diet. Isobutyric acid was also not affected by the enzyme in hens fed 0% HB-based diets. In contrast, isobutyric acid was lower for the 0 compared to 0.01 and 0.1% BGase in 73% HB diets. The isovaleric acid percentage was higher for 0.01 than 0.1% BGase at the 0% HB, whereas an enzyme effect was not found for hens fed 73% HB. The propionic acid percentage was higher for 0 than 73% HB (21.6 vs. 21.2%). Further, the propionic acid percentage was affected by BGase with the ranking from high to low being 0, 0.1 and 0.01% (21.9, 21.4, and 20.9%). The percentage of valeric acid was lower for 0 (1.2%) than 0.01 (3.0%) and 0.1% (2.4%) BGase. The caproic acid percentage was higher for 73% HB fed hens in comparison to those consuming 0% HB diets (1.1 vs. 0.8%), and it was lower for 0 than 0.1% BGase (0.79 vs. 1.3%).

## DISCUSSION

The β-glucan molecular weight distribution of ileal digesta was analyzed to investigate if exogenous BGase depolymerizes high molecular weight β-glucan into lower molecular weight carbohydrates, which were hypothesized to provide increased fermentable substrates for microbes in the lower gastrointestinal tract of laying hens. Beta-glucan Mw and MW-10% were higher with 73% HB than 0% HB diets, which agrees with previous research (Cui et al., 2000; Karunaratne et al., 2021a). Further, in broiler chickens overall β-glucan molecular weight in the ileal digesta (birds fed 60% HB-based diets) was approximately 10 times lower compared with the β-glucan molecular weight found in the 60% HB diets without the addition of BGase (Karunaratne et al., 2021a), although it has not been analyzed in the current laying hen research. This difference between diet and digesta may be associated with the activation of endogenous BGase in HB due to the moistening of grain when interacting with the digestive tract secretions (Ribeiro et al., 2011). Further, the activity of microbial BGase in the upper gastrointestinal tract also affects the depolymerization of β-glucan in chickens (Józefiak et al., 2006; Cardoso et al., 2014). Barley cell walls may release β-glucan molecules associated with other non-starch polysaccharides, including heteroxylans, as well as protein and phenolic acids (Burton and Fincher, 2014) with the exposure to enzymes and secretions in the chicken gastrointestinal tract, and contribute to the reduction of β-glucan molecular weight. In addition, dietary BGase further reduced molecular weight parameters, confirming BGase mediated depolymerization of β-glucan in the ileal digesta of laying hens. Moreover, the BGase mediated reduction of MW-10% indicates an increased proportion of low molecular weight β-glucan in ileal digesta, which might increase carbohydrate fermentation in laying hens. Further, MW-10% is made up of a proportion of oligosaccharides when high levels of BGase (0.1%) is used in the diets. However, the proportion of oligosaccharides cannot be quantified in the current research since the analyzed molecular weight parameters were qualitative but not quantitative. It has been reported that high molecular weight β-glucan increased colonic SCFA compared to low molecular weight β-glucan in pigs (Luo et al., 2019). It might be due to the increased fermentation of low molecular weight β-glucan in the small intestine as β-glucan is primarily digested in the small intestine, and only the high molecular weight β-glucan is available for the fermentation in the colon of pigs. However, data regarding β-glucan fermentation are not available in chickens, which have a comparatively lower capacity for small intestinal fermentation. Of note, BGase reduced molecular weight parameters only when hens were fed diets based on 73% HB, but not those based on 0% HB, which may be due to structural differences between HB and wheat β-glucan. Beta-glucan in cereal grains predominantly yields low degrees of polymerization when exposed to lichenase, and results in tri- or tetra-saccharides of (1-3),(1-4)-β-D-glucan, which is often referred to as the DP3: DP4 ratio (the ratio of 1-3 to 1-4 β-glycosidic bonds). High or low DP3: DP4 ratios increase the predominant molar proportion (tri- or tetra-saccharides) and result in cereal β-glucan with less water-solubility and high gelling ability (Burton and Fincher, 2014). The more uniform structure resulted from a higher predominant molar proportion leads to increased β-glucan aggregation and, in turn, lower solubility and susceptibility to BGase (Karunaratne and Classen, 2019).

Cereal non-starch polysaccharides, including arabinoxylan and β-glucan, consist of both insoluble and soluble components. High molecular weight insoluble non-starch polysaccharides in the cell wall are released and solubilized in the digestive tract by non-starch polysaccharidases (endo-β-glucanases in the case of β-glucan). The enzymes hydrolyze the ingested and newly generated high molecular weight soluble non-starch polysaccharides into low molecular weight soluble non-starch polysaccharides in the digestive tract of chickens (Bautil et al., 2019). Size exclusion chromatography followed by Calcofluor post-column detection only measures the soluble fraction of cereal β-glucan at a point in time, although both insoluble and soluble β-glucan is available and fluctuates in the digesta. The addition of BGase to the HB-based diets of laying hens did not show a bimodal molecular weight distribution curve, including the separate high molecular weight peak (in addition to Mp), as observed in broiler chickens (Karunaratne et al., 2021a). It might be associated with the difference in the microbial community in the digestive tract of laying hens compared to broilers. It is possible that the more mature gut microbiota contains more diverse β-glucanase activity, which degrades high molecular weight β-glucan as quickly as it is released from the cell walls. The more fermentable β-glucan fraction might have been utilized in the upper digestive tract by the more mature gut microbiota of laying hens, although low molecular weight β-glucan is found in the ileum.

Beta-glucanase mediated ileal β-glucan molecular weight distribution should be related to ileal viscosity since the increasing dose of BGase breaks down high molecular weight β-glucan into lower molecular weight carbohydrates that are less viscous. The molecular weight of soluble carbohydrates affects the digesta viscosity of broiler chickens (Bedford and Classen, 1992). However, ileal viscosity was low in 73% HB- compared to 0% HB-based treatments, even though the molecular weight values of ileal digesta and diet TDF levels, including total β-glucan, were higher in HB (Mw 19715; TDF 29.1%; β-glucan 8.03%) in comparison to wheat (Mw 17932; TDF 16.1; β-glucan 0.75). However, other factors also determine the intestinal viscosity in chickens, including the solubility, structure and configuration of non-starch polysaccharides (Boros et al., 1993; Saulnier et al., 1995). Wheat has a higher content of arabinoxylan compared to HB, which may be a factor that increases ileal viscosity when hens were fed 0% HB-based diets (Choct and Annison, 1992; Kiarie et al., 2014). However, the lower ileal viscosity in birds fed 73% HB- in comparison to 0% HB-based diets contrasts with grain viscosity when extracted in Na acetate buffer. The viscosity of the extracted wheat was 1.7 cP, whereas it was not measurable in HB due to extremely high viscosity. These data demonstrate the importance of extraction media when estimating viscosity; in this case, the comparison is between Na acetate buffer and the digestive process. The lower ileal viscosity in the birds fed 73% HB over 0% HB might also be attributed to the competence of ileal microbiota to digest high molecular weight β-glucan but not arabinoxylan.

The concentrations of SCFA in the ileum and ceca of laying hens were higher than the concentrations mentioned in previous laying hen research (Johnson et al., 2008; Taylor et al., 2018), although the procedure of SCFA analysis including the extraction method was similar across the experiments. It might be associated with the difference in the age of laying hens, the composition of the diets and the rearing environment that eventually affects the microbial population in the digestive tract of the birds. The concentrations of total SCFA and acetic, propionic, butyric, caproic, and lactic acids in the ileum decreased with 0.01 and 0.1% BGase when the birds were fed a 73% HB-based diet, indicating reduced fermentation of carbohydrates such as β-glucan and starch. Further, BGase decreased ileal isobutyric and isovaleric acids in the birds given 0 and 73% HB, indicating reduced protein fermentation. Therefore, the reduction of ileal fermentation could be attributed to the reduced fermentable nutrients due to increased nutrient digestibility caused by dietary BGase (Choct et al., 1996). Unfortunately, this suggestion cannot be confirmed because digestibility of key nutrients was not measured in the current research. However, BGase decreased the molecular weight of β-glucan in the ileal digesta, and the content of β-glucan did not decrease with the addition of the enzyme to the diets, which is contradictory to the above speculation. In addition, BGase did not affect the concentrations of major SCFA in the ileum of broiler chickens given 0% HB diets, as noted earlier (Karunaratne et al., 2021b). It supports the results of Choct et al. (1999), who found that the addition of xylanase to a wheat-based diet decreased ileal SCFA compared to the control broilers fed a wheat-based diet without enzyme. It might also be associated with the increased feed passage rate as a result of the reduction of ileal viscosity by non-starch polysaccharidases (Lazaro et al., 2003b), and thereby low molecular weight β-glucan passing rapidly into the ceca. As a result, there would be less fermentable substrate available for fermentation in the ileum. If this concept is correct, carbohydrate fermentation should increase in the ceca. However, the concentrations of acetic and propionic acids in the ceca decreased with 0.1% BGase when the birds were fed 73% HB-based diets, and isobutyric, valeric, and caproic acid concentrations increased with the BGase addition. In previous research, BGase affected cecal SCFA with less clear trends, except for 0.1% BGase increased all SCFA compared to 0.01% BGase in the broilers given 60% HB-based diets (Karunaratne et al., 2021a). An important question is whether SCFA levels are an accurate predictor of carbohydrate fermentation. The concentration of SCFA does not only depend on the availability of fermentable substrates and microbial fermentation rates but also other factors, including inflow, outflow, absorption, mean retention time and microbial degradation of SCFA (Macfarlane and Macfarlane, 1997). Moreover, the actual rates of production of SCFA are impossible to obtain in poultry due to the problems associated with cecal portal vein catheterization. The SCFA content in digesta varies with sample collection time since the variability of the ileal and cecal evacuation of the chickens, although the sample collections were designed to avoid treatment bias.

The ileal and cecal pH of laying hens were not affected by the treatment, therefore not related to SCFA concentrations in the current research. However, dietary BGase consistently increased ileal pH while reducing cecal pH in broiler chickens given similar diets (Karunaratne et al., 2021a), which suggested that microbial fermentation has shifted from the ileum to ceca with the addition of BGase to the diets. These differences in response to BGase might be associated with the differences in the age and diet composition of broiler chickens compared with laying hens, all of which likely affects the structure and density of the microbial population in the digestive tract.

The estimated and analyzed enzyme activity of BGase were not exactly similar in the current study. The 0% BGase diets contained 8,938 BU/kg BGase activity. It might be due to the presence of endogenous BGase in hulless barley, which is known to be present in grain (Cardoso et al., 2014). Diet β-glucan molecular weight has been reduced to a larger degree in the ileal digesta (Mp from 762 × 10^3^ to 78 × 10^3^; Mw from 648 × 10^3^ to 80 × 10^3^) of broiler chickens fed 60% hulless barley-based diets even without the addition of dietary BGase in a previous study that was completed by our lab (Karunaratne et al., 2021a). Further, the same broiler chicken study observed similarly high BGase activity (16,267 BU/kg) in the 0% BGase diets. The 0.01 and 0.1% BGase diets had less than the estimated BGase activity levels, which could be related to moderate pellet conditioner temperature (68–70°C for 10–12 min) induced enzyme damage, and it is supported by the similar recovery of approximately 36.6 ([16,267−8,938]/20,000 × 100) and 42.1% ([93,092−8,938]/200,000 × 100) of added enzyme activity for the 0.01 and 0.1% treatments, respectively. However, the objective of using 3 different dietary BGase levels was to study the effect of BGase level on the depolymerization of ileal soluble β-glucan and carbohydrate fermentation in the ileum and ceca of laying hens. The enzyme level affected the ileal soluble β-glucan depolymerization despite the lower than the expected enzyme activity values in the diets. Therefore, the effect of the enzyme activity difference between analyzed and expected values is minimum on the overall results of the current study. Further, xylanase activity was nondetectable in the diets, and according to the literature, this is the first time a purified BGase has been used in laying hen feed containing barley. Therefore, the current study directly demonstrates the single effect of exogenous BGase on β-glucan molecular weight in the terminal small intestine of laying hens.

Exogenous BGase depolymerized ileal digesta soluble β-glucan in the laying hens, while ileal β-glucan concentration is not affected by the enzyme addition to the diets. However, the utilization of β-glucan is not apparent based on the current research findings since the lack of data regarding β-glucan digestibility in the ileum. The ileal fermentation decreased with the dietary inclusion of BGase, whereas cecal fermentation is not affected by the enzyme use despite the increasing availability of potentially fermentable low molecular weight β-glucan in the ileum. It is difficult to relate the BGase mediated ileal soluble β-glucan molecular weight changes to the fermentation due to the absence of a major shift in SCFA concentrations or digestive tract pH in the laying hens.

It is concluded that exogenous BGase depolymerized high molecular weight soluble hulless barley β-glucan by the time digesta reached the ileum and resulted in a higher proportion of low molecular weight β-glucan. In addition, there was an enzyme dosage effect on β-glucan depolymerization in laying hens. Despite the changes in ileal digesta soluble β-glucan molecular weight, the effects of BGase on fermentation indicators (short chain fatty acid levels, pH) were relatively minor, except the decreased concentrations of major short chain fatty acids in the ileum.
